# Adsorptive Removal of Hexavalent Chromium by Diphenylcarbazide-Grafted *Macadamia* Nutshell Powder

**DOI:** 10.1155/2018/6171906

**Published:** 2018-04-19

**Authors:** Londolani C. Maremeni, Sekomeng J. Modise, Fanyana M. Mtunzi, Michael J. Klink, Vusumzi E. Pakade

**Affiliations:** ^1^Department of Chemistry, Faculty of Applied and Computer Sciences, Vaal University of Technology, Private Bag X021, Vanderbijlpark 1911, South Africa; ^2^Institute of Chemical and Biotechnology, Faculty of Applied and Computer Sciences, Vaal University of Technology, Vanderbijlpark 1911, South Africa

## Abstract

*Macadamia* nutshell powder oxidized by hydrogen peroxide solutions (MHP) was functionalized by immobilizing 1,5′-diphenylcarbazide (DPC) on its surface. The effectiveness of grafting was confirmed by the Fourier transform infrared spectrum due to the presence of NH and C=C stretches at 3361, 1591, and 1486 cm^−1^, respectively, on the grafted material which were absent in the nongrafted material. Thermogravimetric analysis revealed that the presence of DPC on the surface of *Macadamia* shells lowered the thermal stability from 300°C to about 180°C owing to the volatile nature of DPC. Surface roughness as a result of grafting was appreciated on the scanning electron microscopy images. Parameters influencing the adsorptive removal of Cr(VI) were examined and found to be optimal at pH 2, 120 min, 150 mg/L, and 2.5 g/L. Grafting MHP with DPC leads to an increase in the Langmuir monolayer capacity from 37.74 to 72.12 mg/g. Grafting MHP with DPC produced adsorbent with improved removal efficiency for Cr(VI).

## 1. Introduction

Natural biosorbents like mango kernels, *Macadamia* nutshells, coconut shells, pine cone, almond shells, sawdust, palm branches, and hazelnut consist in their plant cell walls chiefly the lignin, cellulose, and hemicellulose as the main structural components [1–5]. Consequently, these materials contain a cornucopia of surface functional groups including ketones, aldehydes, esters, ethers, and alcohols. The escalating use of natural biosorbents in adsorption for the remediation of metal pollutants has been largely due to their abundant availability, inexpensiveness, biodegradability, easy desorption, good reusability, and the diverse functional groups they possess which are needed for metal abstraction [6–8]. Further, these materials are regarded as cheap because they require minimum processing. However, when used as adsorbents, some shortfalls like poor surface areas and low adsorption capacities are experienced probably because of the highly cross-linked polymeric nature of the said materials [1, 4, 9]. In addition, upon being used as adsorbents, there is a tendency of small organic molecules trapped within the polymeric cross-linked chains being released into the environments causing high biological oxygen demand and chemical oxygen demand [9]. These drawbacks have restricted the utilization of biosorbents in adsorption in their pristine form.

To address the downfalls, researchers have explored various pretreatment techniques such as (i) biological treatment with fungi or bacteria, (ii) chemical methods using acid, alkaline, oxidation, or solvent extraction, (iii) physical methods through sonication, pyrolysis, or mechanical agitation, and (iv) physicochemical methods, for example, steam explosion [5, 10]. During pretreatment, surface chemical properties of an adsorbent could be altered through masking or elimination of certain groups or exposure to more adsorption sites [7, 11]. Greater separation of components is achieved much faster and conveniently with chemical pretreatment methods than biological methods [5]. Depending on the intended use of the biomass and the properties desired, various chemical agents, H_2_O_2_ [7], ozone [12], H_3_PO_4_ [13], HNO_3_ [14], NaOH [4], and so on, have been reconnoitred for either hydrolysis, oxidation, or delignification [5]. Oxidative chemical treatment could lead to increased oxygenated functionalities like COOH, C–O–C, CO, and OH which may then increase metal ion complexation, ion exchange, and chelation [5, 15].

To supplement pretreatment methods for improved performance, further modifications (grafting, cross-linking, or polymerization) could be carried out on the surface of adsorbents. Acrylic acid monomer units were grafted on the surface of coir pith, and an adsorption capacity increase from 165 to 196 mg/g was reported [16]. The improved performance was attributed to the increased density of –COOH adsorption sites on the material surface. Behbahani et al. [17] grafted 1,5′-diphenylcarbazide on the surface of multiwalled carbon nanotubes for the extraction of Cd(II) ions from water samples and food products. Extraction efficiency greater than 97% with a limit of detection (LOD) of 0.05 ng/mL was achieved. Grafting or polymerization introduces specific functional groups, NH, SH, OH, or COOH, on the surface of adsorbents. In a recent study [18], it was shown that modification of natural *Populus tremula* fibers with amino ligands led to increased adsorption capacity of acid blue 25 from 22.33 mg/g for unmodified to 48 mg/g for hydrazine fiber and 67 mg/g for ethylenediamine fiber. Masau stones were chemically modified with diethylenetriamine through a cross-linking protocol, and 87.32 mg/g adsorption capacity was reported [19]. The presence of protonated amino groups at acidic conditions was credited to the biosorption-coupled reduction removal mechanism of Cr(VI). Similarly, Pakade et al. [20] achieved high adsorption capacity of 145.5 mg/g following modification of *Macadamia* nutshell-based carbon with diethylenetriamine and triethylamine ligands. From the mentioned studies, it is obvious that Cr(VI) has a high affinity for amine functional groups, and it is this narrative that led to the present study where *Macadamia* nutshells were functionalized, for the first time, with 1,5′-diphenylcarbazide for the eradication of hexavalent chromium from aqueous solution.

Hexavalent chromium [Cr(VI)] and trivalent chromium [Cr(III)] are the most stable forms of Cr in the environment. Cr(VI) is a toxic strong oxidant capable of penetrating the biological cell membranes due to its similarity to sulphates in structure [21]. On the other hand, Cr(III) is less toxic and is regarded as a micronutrient at minute concentrations but could be toxic if concentrations are high [22]. Exposure to Cr(VI) compounds may lead to health detrimental effects like lung cancer, kidney damage, epigastric pain, nausea, vomiting, and even death [23, 24]. Careless disposal and improper treatment of effluents from leather tanning, electroplating, textile dyeing, mining, and wood preservation industries lead to contamination of the environment by Cr(VI) compounds. Owing to their advantages of simplicity, low cost, and fine-tuning of functional groups, adsorption methods have found more applications for the elimination of toxic metals. Therefore, the current study aimed to employ *Macadamia* nutshells oxidized with H_2_O_2_ in removing Cr(VI) from aqueous solution and also investigate whether any amelioration in adsorption performance would be achieved following functionalization of H_2_O_2_-treated materials with 1,5′-diphenylcarbazide. Confirmation of the grafting was sought after using the Fourier transform infrared spectroscopy, thermal analysis, and scanning electron microscopy. Adsorption was evaluated by varying pH, time, concentration, and dosage, and various models were used to substantiate the adsorption mechanism involved.

## 2. Materials and Methods

### 2.1. Chemicals and Instrumentation

Reagent grade potassium dichromate, stannous chloride, ethyl chloride, methanol, toluene, hydrogen peroxide (50% wt.), sodium hydroxide, hydrochloric acid, and 1,5′-diphenylcarbazide were purchased from LabChem and Merck Chemical Co. (Johannesburg, South Africa) and were used without further purification. pH adjustments were monitored by HI 2210 from Hanna Instruments (Johannesburg, South Africa), while ultrapure water used for all reactions was obtained from Siemens LaboStar equipment (Warrendale, Pennsylvania, USA). Batch adsorption experiments were conducted in duplicate on an end-over-end Labcon 3100U electrical shaker (Maraisburg, South Africa). Sample preparation and analysis of chromium in solution before and after adsorption was conducted as detailed in our previous studies [4]. *Macadamia* nutshells were kindly donated by Eastern Produce Estates SA (Pty) Ltd from Tzaneen and Danroc (Pty) Ltd from Barbaton, South Africa.

### 2.2. Preparation of Adsorbents

#### 2.2.1. Chemical Pretreatment

Following removal of debris and dirt, *Macadamia* nutshells were washed with running tap water and then dried for 24 h in an oven at 105°C. The dried nutshells were then ground, milled, and sieved collecting between 90 *µ*m and 150 *µ*m screens. The collected sample was designated as raw *Macadamia* nutshells (RMNs). Then, the RMN was oxidized and/or bleached with 20%, 35%, and 50% (v/v) H_2_O_2_ solution, resulting in the formation of more oxygen-bearing functional groups on its surface. The resultant materials were labeled 20 MHP, 35 MHP, and 50 MHP to correspond to the different H_2_O_2_ concentrations used for oxidation of *Macadamia* nutshell powder. The milling process not only aids with particle size reduction but also could reduce the crystallinity and the degree of polymerization of the polysaccharides in biomass [5] leading to improved adsorption performance.

#### 2.2.2. Functionalization of MHP with Diphenylcarbazide

**Figure 1:**
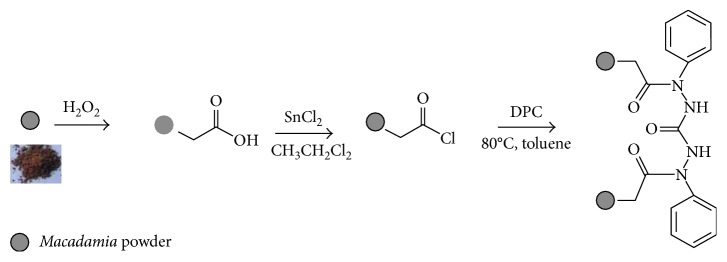
Preparation of diphenylcarbazide-grafted adsorbents.

A method adapted from Behbahani et al. [17] was used with modifications. Briefly, one gram of 20 MHP was suspended in 100 mL of dry ethyl chloride (CH_3_CH_2_Cl_2_) under nitrogen (N_2_) atmosphere in a 250 mL round-neck flask equipped with a magnetic stirrer and a condenser. About 2 g of stannous chloride was added to the mixture. Following 24 h of reflux, the solvent (CH_3_CH_2_Cl_2_) was removed under reduced pressure and the resultant solid was suspended in toluene. About 5 g of excess diphenylcarbazide (DPC) was added to the reaction vessel, and the contents were refluxed for further 24 h. On completion, the solid was filtered, washed with toluene and methanol, and then dried at room temperature. The product was labeled 20 MHPD to signify DPC grafting on 20 MHP. The same procedure was followed but 20 MHP was replaced with 35 MHP and 50 MHP with the resolution products labeled 35 MHPD and 50 MHPD, respectively.  Figure 1 shows the preparation procedure.

### 2.3. Characterization of Adsorbents

Scanning electron microscopic images were obtained from an FEI Quanta 200 SEM (FEI, Hillsboro, OR, USA) and Jeol IT-300 tungsten scanning electron microscopy equipped with secondary backscattering electron detectors and an Oxford energy dispersive X-ray (EDX) analysis external probe. Samples were coated with gold using a Quorum Q150R sputter coater (Quorum Technologies Ltd, East Sussex, UK). The coating thickness was precisely controlled at 5 nm using the film thickness monitor option of the Quorum Q150R sputter. Functional group analysis on the surface of adsorbents was obtained from a PerkinElmer Spectrum 400 FT-IR/FT-NIR spectrometer (Waltham, MA, USA) recording from 4000 to 500 cm^−1^. Thermal analysis of adsorbents was elucidated using a PerkinElmer TGA 4000 thermogravimetric analyzer (Waltham, USA). Centrifugation was accomplished employing a CL10 ThermoScientific centrifuge (Johannesburg, South Africa).

### 2.4. Adsorption Studies

The *Macadamia* adsorbents (MHP and MHPD) were evaluated for their Cr(VI) removal efficacy through batch experiments conducted in duplicate. Various parameters that influence adsorption including the effect of pH (pH 1 to pH 12), contact time (20–180 min), initial adsorbate concentration (25 to 150 mg/L), and adsorbent dosage (0.63 to 10.63 g/L) were studied. In a typical experiment setup, 0.05 g of adsorbent was charged to a solution of Cr(VI) (20 mL of 100 mg/L) contained in a 100 mL glass bottle, and the pH was adjusted with HCl or NaOH diluted solutions. The contents were allowed to react for 120 min on the electrical shaker followed by solid/liquid separation through centrifugation. The concentration of Cr(VI) and total Cr was measured with UV-Vis spectrophotometer T80^+^ (PG Instruments) and atomic absorption spectroscopy (AA-7000 from Shimadzu, Kyoto, Japan), respectively, as detailed in our previous studies [4, 25]. The performance of adsorption was evaluated by calculating the removal percentage (%*R*) and maximum adsorption capacity (*q*_e_ (mg/g)) using (1) and (2), respectively:

(1) $$\begin{array}{c} \%R=\frac{\left(C_{0}-C_{e}\right)}{C_{0}}\times 100, \end{array}$$

(2) $$\begin{array}{c} q_{e}=\frac{V\left(C_{0}-C_{e}\right)}{m}, \end{array}$$

where *C*_e_ is the adsorbate equilibrium concentration (mg/L), *C*_0_ is the adsorbate initial concentration (mg/L), *m* is the mass of adsorbent (g), and *V* is the volume of solution used in adsorption (L).

## 3. Results and Discussion

### 3.1. Characterization of Materials

#### 3.1.1. Fourier Transform Infrared Spectroscopy (FTIR) Analysis

**Figure 2:**
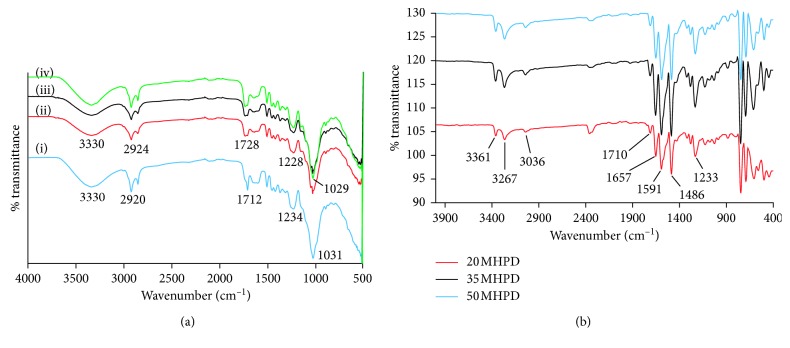
(a) FTIR spectra of RMN (i), 20 MHP (ii), 35 MHP (iii), and 50 MHP (iv) adsorbents. (b) FTIR spectra of 20 MHPD, 35 MHPD, and 50 MHPD adsorbents.

Figure 2(a) depicts the Fourier transform infrared spectroscopy (FTIR) spectra of RMN, 20 MHP, 35 MHP, and 50 MHP adsorbents. The hydrogen peroxide-oxidized *Macadamia* nutshells (MHP) have a broad vibrational stretch at 3330 cm^−1^ accredited to the presence of bonded OH groups of the cellulose, C=O of carboxyl functional groups at 1728 cm^−1^, and the –C–O stretch of the primary alcohol at 1029 cm^−1^. The H_2_O_2_ treatment was mild as the structural backbone of the RMN was not greatly altered, but peak shift characteristics of treatment were observed. In accordance with the results reported during chemical treatment of grape peelings with H_2_O_2_ [7] and lightweight expanded clay aggregate adsorbent with aqueous solution of magnesium chloride and hydrogen peroxide [26], no new peaks were observed suggesting a minimal influence of H_2_O_2_ on the chemical structure of biosorbents. The C=O band at 1712 cm^−1^ in the RMN shifted to 1728 cm^−1^ in MHP adsorbents accompanied by a change in peak shape implying that the H_2_O_2_ yielded C=O of a different chemical environment to the RMN. In addition, the C–O band of primary alcohols also shifted from 1031 to 1029 cm^−1^ after peroxide treatment. The –C–O–C vibrations shifted from 1234 to 1228 cm^−1^ after chemical treatment of the RMN with H_2_O_2_ [27]. Immobilization of DPC on MHP resulted in adsorbents with different functional groups as depicted in Figure 2(b). New absorption peaks at 3361 cm^−1^ attributed to the –NH stretch of DPC, –C=O of the amide at 1657 cm^−1^, –CH stretch of the aromatic ring at 3036 cm^−1^, –CN stretch at 1233 cm^−1^, –CH bending at 743 cm^−1^, and –C=C vibrations of the benzene ring at 1591 and 1486 cm^−1^ were observed. In addition, absorption peaks due to the parent material were still present: carboxyl –C=O at 1710 cm^−1^ and primary alcohol –C–O stretch at 1100 cm^−1^ [18]. All these observations pointed to the effectiveness of modification and immobilization of the DPC ligand.

#### 3.1.2. Thermogravimetric Analysis (TGA) and Differential Thermal Analysis (DTA)

**Figure 3:**
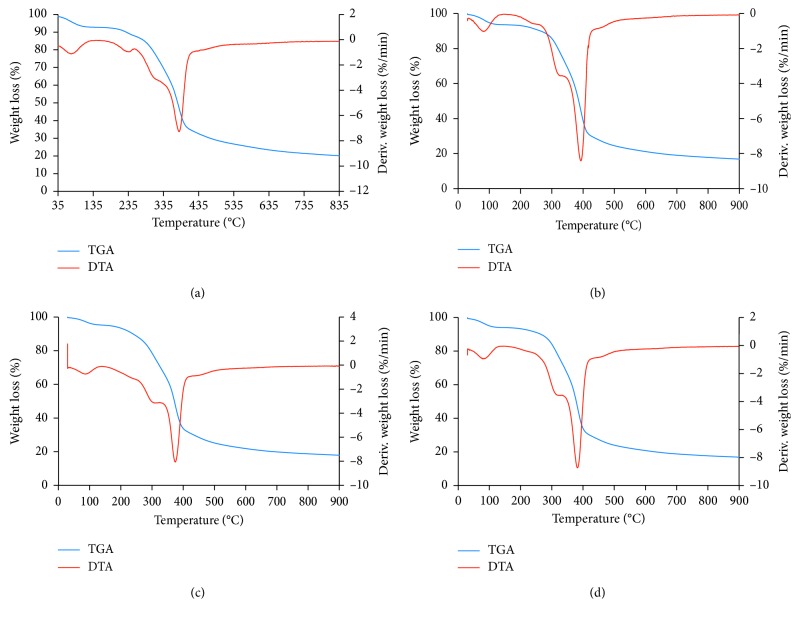
(a)TGA and DTA thermograms for RMN (a), 20 MHP (b), 35 MHP (c), and 50 MHP (d) adsorbents.

Figure 3 shows the TGA and DTA thermograms for the RMN and MHP. According to Paduraru et al. [28], thermal degradation of biomass follows four distinct stages, namely, the moisture evolution and decomposition of hemicellulose, cellulose, and lignin. These different decomposition stages were also observed with our materials. In all thermograms, the first decomposition observed from 50°C to 90°C was attributed to the loss of moisture and sorbed water. The second decomposition with the maximum mass loss rate at 318°C, 303°C, 326°C, and 317°C for RMN, 20 MHP, 35 MHP, and 50 MHP, respectively, was due to degradation of hemicellulose structures. The third decomposition observed at 385°C (RMN), 392°C (20 MHP), 374°C (35 MHP), and 382°C (50 MHP) was accredited to cellulose structure disintegration. The final degradation which took place beyond hemicellulose degradation was attributed to the slow decomposition of lignin. In addition, the RMN, 20 MHP, and 35 MHP displayed loss of volatile components, probably CH_4_, H_2_, CO_2_, or CO, at about 250°C, and this was absent in 50 MHP adsorbent probably because the 50% (v/v) H_2_O_2_ treatment was too harsh and eliminated all the volatile components Yang et al. [29] also observed evolution of volatile compounds (CH_4_, H_2_, CO_2_, and CO) at temperatures below 300°C during pyrolysis of hemicellulose. The exact degradation temperatures of cellulose and hemicellulose were different in all materials indicating that the treatment influenced the structure and surface properties of these materials.

**Figure 4:**
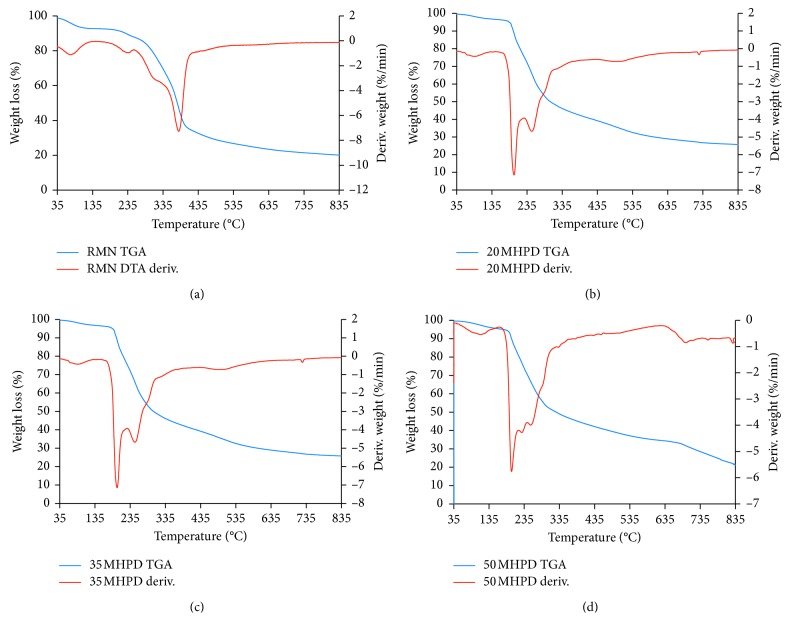
TGA and DTA thermograms for RMN (a), 20 MHPD (b), 35 MHPD (c), and 50 MHPD (d) adsorbents.

The DPC-grafted materials exhibited distinct thermograms compared to the H_2_O_2_-treated materials. The thermograms are shown in Figure 4. In all thermograms, loss of moisture and adsorbed water was observed at about 80°C. The more pronounced degradation at about 205°C was attributed to the decomposition of DPC from MHPD. Another notable peak was at 270°C attributable to the decomposition of cellulosic structures. Slow degradation of lignin can be appreciated from 335°C onwards. Clearly, the 50 MHPD exhibited slightly different decomposition peak shapes. Dhakal et al. [30] alluded that the difference in peak shapes and decomposition temperatures showed that the structural backbone of the adsorbents was affected by the chemical treatment to which they were exposed to.

#### 3.1.3. Scanning Electron Microscopy (SEM)

**Figure 5:**
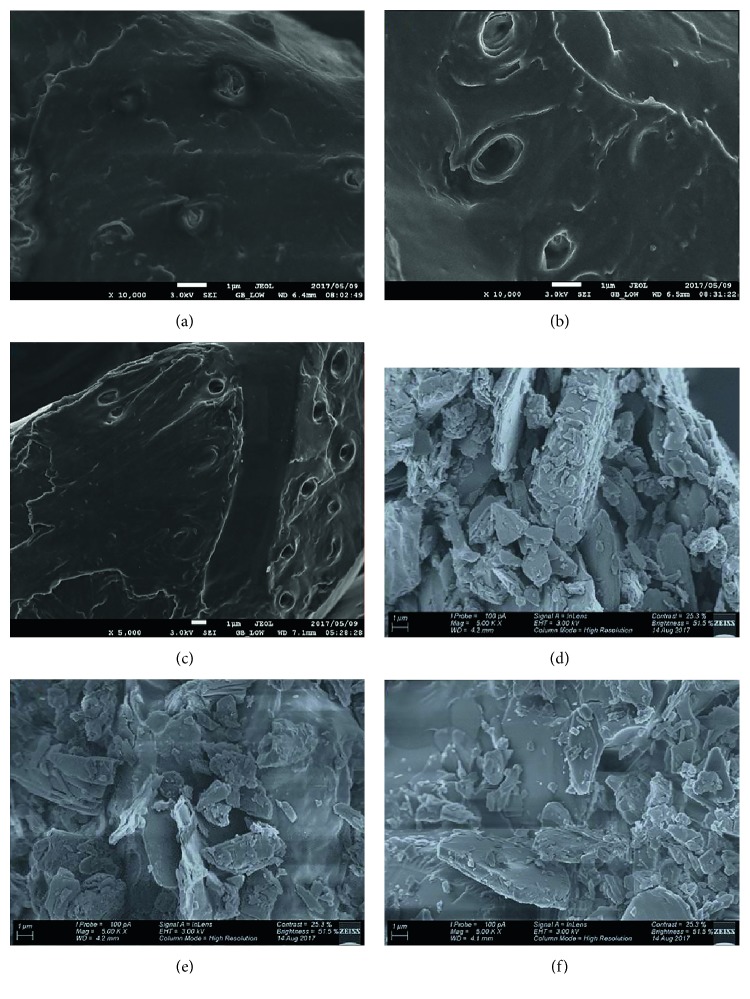
Scanning electron microscopic images for 20 MHP (a), 35 MHP (b), 50 MHP (c), 20 MHPD (d), 35 MHPD (e), and 50 MHPD (f).

The morphological properties of the adsorbent surface were elucidated using scanning electron microscopy (SEM). The SEM images for MHP and MHPD adsorbents are shown in Figure 5. RMN SEM was typical of plant material SEM with rough surface texture and scaling but with no observable porosity [4]. Upon treatment with H_2_O_2_, some surface porosity was observed as round spots in MHP samples. Grafting MHP with DPC yielded materials with much rougher texture, but pores disappeared. The differences in surface topographies between MHP and MHPD adsorbents proved that functionalization did occur. The disappearance of open pores due to grafting was observed elsewhere [31], while Albadarin et al. [19] also noted differences in surface topographies of amine-modified masau stones incurred by chemical treatment.

#### 3.1.4. Brunauer–Emmett–Teller (BET) Analysis

The Brunauer–Emmett–Teller (BET) surface analysis was carried out to assess the pore volume, surface area, and pore size of the adsorbents.  Table 1 lists the BET, surface area, pore volume and pore diameter results for MHP and MHPD adsorbents. Typical of biomass, the surface area values were relatively small ranging from 0.0063 to 0.5093 m^2^/g. It seems that grafting of DPC on MHP decreased the surface areas as in the case of 35 MHP (1.0019 m^2^/g) and 35 MHPD (0.2034 m^2^/g) as a result of surface coverage. On the contrary, the surface area for 20 MHP increased to 0.5093 m^2^/g after DPC grafting. Surface areas in the range of 2.8 to 6.3 m^2^/g for palm branches [32] and from 3.1477 to 3.6672 m^2^/g for peat and coconut fibers [33] have been reported.

### 3.2. Adsorption Experiments

#### 3.2.1. Effect of pH

**Figure 6:**
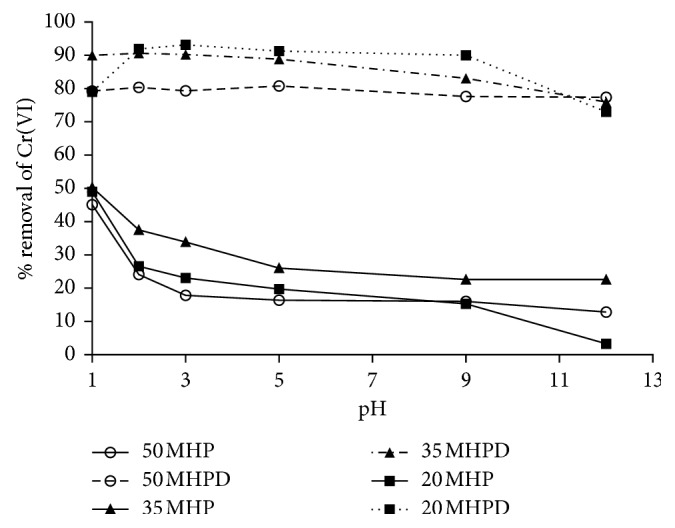
Effect of initial solution pH on the removal of Cr(VI) by MHP and MHPD adsorbents (conditions: initial Cr(VI) concentration 150 mg/L; dosage 2.5 g/L; and time 120 min).

The effect of pH on the removal of hexavalent chromium by 20 MHP, 35 MHP, 50 MHP, 20 MHPD, 35 MHPD, and 50 MHPD was carried out in batch adsorption experiments by varying the pH from 1 to 12 while the initial concentration of Cr(VI) was 150 mg/L and the adsorbent dose was 2.5 g/L. The results are displayed as %*R* versus initial pH in Figure 6. Both the MHP and the MHPD adsorbents exhibited a similar trend where %*R* decreased as the initial pH of the solution was increased from 1 to 12. Highest removal of Cr(VI) took place at pH 1, but there was a clear separation in terms of performance between the MHP and MHPD adsorbents, with the latter exhibiting superior removal starting at 90% and decreasing to 79%, while the former ranged from 50% to 4% removal. That is, all these adsorbents showed a strong dependence in solution pH where maximum adsorption occurred at acidic conditions [4, 34]. The high removal at low pH is the result of electrostatic attraction between positively charged adsorbent sites (protonation) (COOH_2_^+^ and OH_2_^+^ for MHP and NH^+^ for MHPD) and the hydrogen chromate ions [35]. Hydrogen chromate (HCrO_4_^−^) is usually the most dominant Cr(VI) species at acidic conditions and under oxidizing conditions. On the other hand, dichromate (Cr_2_O_7_^2−^) is stable at weakly acidic and low oxidizing conditions, but as the pH is increased towards the basic region, the equilibrium tends to shift to chromate (CrO_4_^2−^) [25, 32]. The decline in %*R* as the pH was increased could be due to the depletion of protons resulting in less protonated sites and more OH^−^ groups that can lead to competition with chromate ions for adsorption sites. Similar pH efficiency trends have been reported by several researchers [20, 31, 35–37]. Besides the predicted electrostatic attraction of Cr(VI) to the cationic group mechanism, the reduction of Cr(VI) to Cr(III) cannot be ruled out because it has been demonstrated in the past that *Macadamia* nutshell-based adsorbents favored adsorption-coupled reduction uptake of Cr(VI) [4, 38].

#### 3.2.2. Effect of Contact Time and Kinetics

**Figure 7:**
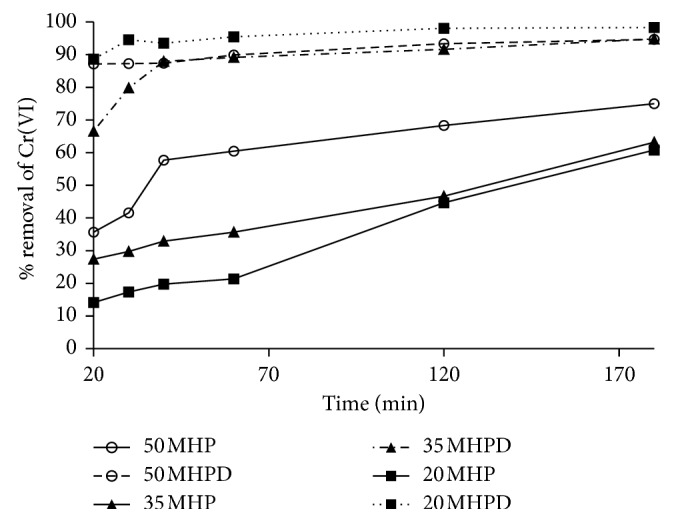
Effect of contact time for the removal of Cr(VI) by MHP and MHPD adsorbents (conditions: initial Cr(VI) concentration 150 mg/L; dosage 2.5 g/L; and pH 1).

To investigate the feasibility and efficiency of Cr(VI) adsorption onto MHP and MHPD adsorbents, contact time was varied from 20 to 180 min, while all other parameters were kept constant. The %removal of Cr(VI) by MHP and MHPD as a function of time is shown in Figure 7. Two distinct sets of results were observed where the MHP adsorbents performed poorly compared to MHPD. The %*R* for MHP adsorbents increased from 12 to 70% as time was varied from 20 to 180 min and seemed to not have reached equilibrium even after 180 min of contact time. This could mean that the process of adsorption by MHP adsorbents was quite slow as there were adsorption sites not accessible after 180 min of reaction time. The order of adsorption efficiency was 50 MHP > 35 MHP > 20 MHP. Grafting MHP with DPC yielded adsorbents with better performance in terms of %*R* and reaction kinetics. The %*R* of MHPD was much higher than that of MHP ranging from 65 to 98%, while the saturation was achieved only after 40 min. In some of these curves, the three stages of adsorption processes were notable, that is, the boundary layer saturation (20 to 30 min), diffusion into internal pores (30 to 40 min), and equilibration (beyond 40 min). The high affinity of the adsorbate to adsorption sites (physical adsorption or ion exchange) was associated with the higher sorption rate at initial times [34]. MHPD adsorbents possessed more functional groups with better accessibility due to the high affinity of Cr(VI) for protonated amino groups. The order of efficiency was 20 MHPD > 50 MHPD > 35 MHPD.

#### 3.2.3. Kinetic Parameters

Pseudo-first-order [39] and pseudo-second-order [40] kinetic models were employed to study the kinetic parameters governing the adsorption of Cr(VI) to MHP and MHPD adsorbents. The time dependency data were fitted into nonlinear pseudo-first-order (PFO) and pseudo-second-order (PSO) equations (3) and (4). The results as illustrated in Table 2 revealed that all the adsorbents, except 20 MHP and 35 MHPD, followed the PSO rate model as judged by the higher coefficient of correlation (*R*^2^), lower *X*^2^, and closeness of *q*_t_ to *q*_e_ values in PSO in comparison to the PFO model. It could then be inferred that PSO represented the adsorption of Cr(VI) by all MHP and MHPD adsorbents barring the 20 MHP and 35 MHPD which obeyed PFO. The adsorbents obeying PSO were predicted to favor chemisorption [31, 33], while those described by PFO favored physisorption [18]. It has been argued by Albadarin et al. [19] that the PFO and PSO kinetic models are empirical equations and therefore not fully account for the chemical and physical interactions of adsorbates. The complex nature of Cr(VI) interaction with adsorbents (i.e., adsorption/reduction) makes it difficult to explain the mechanism with only one model as is the case with 20 MHP. The *k* values were relatively small, implying that the sorption process required more time to approach saturation [19].

(3) $$\begin{array}{c} q_{t}=q_{e}\left(1-\exp^{-k}1^{t}\right), \end{array}$$

(4) $$\begin{array}{c} q_{t}=\frac{tk_{2}\cdot q_{e}^{2}}{\left(1+k_{2}tq_{e}\right)}, \end{array}$$

where *k*_1_ (1/min) is the PFO rate constant, *q*_t_ (mg/g) is the amount of Cr(VI) (mg) adsorbed by the adsorbent (g) at time *t*, and *k*_2_ (g/mg min) is the PSO rate constant.

#### 3.2.4. Intraparticle and Liquid Film Diffusion

**Figure 8:**
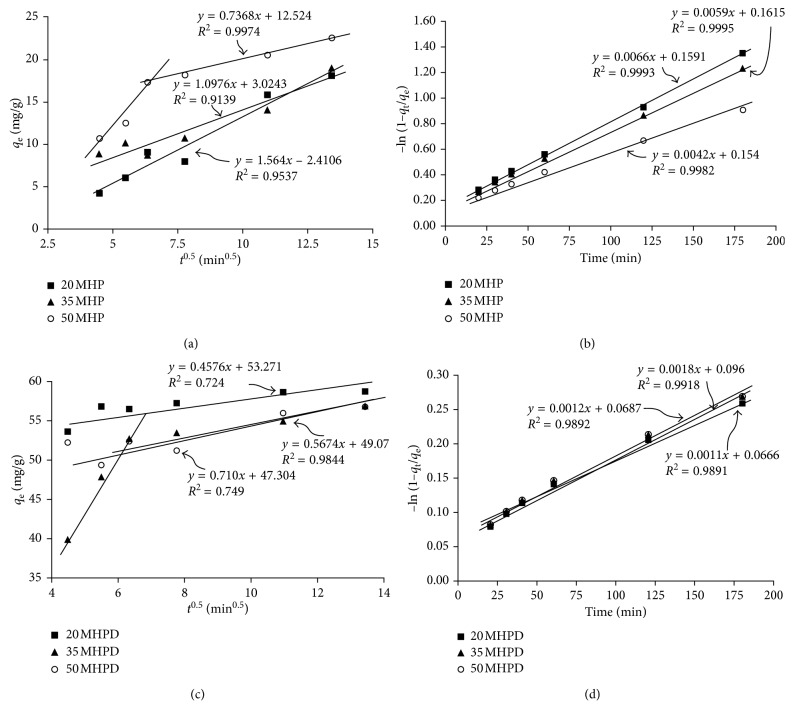
Intraparticle and liquid film diffusion of MHP (a, b) and MHPD (c, d) adsorbents.

Intraparticle and/or film diffusion (external mass transfer) represent possible mechanisms by which adsorption of adsorbates onto porous materials could take place [41]. The intraparticle diffusion model proposed by Weber et al. [42] represented here by (5) was used to determine the rate-controlling steps and mechanism of adsorption of Cr(VI). In addition, liquid film diffusion was evaluated by fitting the data into (6).

(5) $$\begin{array}{c} q_{t}=k_{d}t^{0.5}+C, \end{array}$$

(6) $$\begin{array}{c} \text{Ln}\left(1-\frac{q_{t}}{q_{e}}\right)=-k_{\mathrm{fd}}t, \end{array}$$

where *k*_d_ (mg/(g·min^0.5^)) is the rate constant for intraparticle diffusion, *k*_fd_ is the film diffusion rate constant, and the boundary layer thickness is represented by the intercept *C* (mg/g). According to literature [43–45], intraparticle diffusion is deemed the only rate-controlling step when the plots of *q*_t_ versus *t*^0.5^ yield a straight line passing through the origin. It can be seen in Figures 8(a) and 8(c) that the adsorbents exhibited different mechanisms, that is, linear line over the entire time interval (20 MHP, 35 MHP, 20 MHPD, and 50 MHPD) and multilinearity (50 MHP and 35 MHPD). Even those that were straight lines, none passed through the origin but implied the dominance of the intraparticle diffusion mechanism and insignificant mass transfer resistance. The curves with multilinearity showed the existence of film diffusion (the first steep curve) and intraparticle diffusion (the second flat curve). Assessment of the liquid thin-film diffusion model (Figures 8(b) and 8(d)) shows great linearity with *R*^2^ > 0.98, but lines did not pass through the origin point suggesting that film diffusion was also not the only rate-limiting step.

#### 3.2.5. Effect of Initial Concentration and Adsorption Isotherms

**Figure 9:**
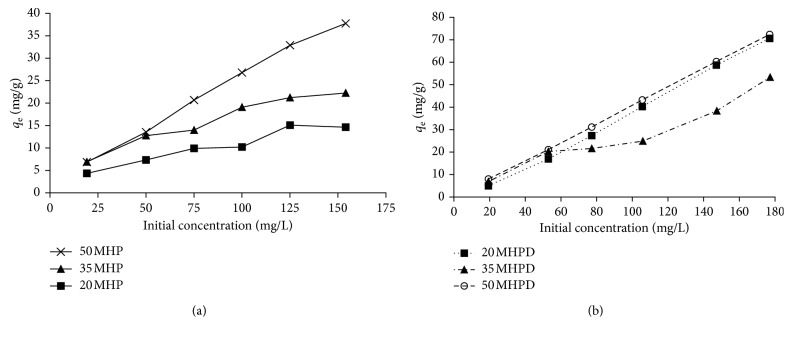
Effect of initial solution concentration for the removal of Cr(VI) by MHP and MHPD adsorbents (conditions: contact time 180 min; dosage 2.5 g/L; and pH 2).

Figure 9 shows the adsorption capacity (*q*_e_) as a function of initial adsorbate concentration. It can be appreciated that the adsorption capacities increased as the initial concentration of Cr(VI) was increased from 20 to 180 mg/L. The magnitudes of *q*_e_ varied as follows: 4.9 to 15 mg/g for 20 MHP, 5.1 to 20 mg/g for 35 MHP, 5.1 to 40 mg/g for 50 MHP, 6 to 70 mg/g for 20 MHPD, 7 to 50 mg/g for 35 MHPD, and 7 to 74 mg/g for 50 MHPD. These results clearly showed that the adsorption of Cr(VI) was concentration dependent, that no saturation was realized except 20 MHP and 35 MHP, and that MHPD adsorbents were superior to MHP adsorbents. The increase in sorption capacity as concentration was increased could be explained on the basis that, at low adsorbate concentrations, the number of available adsorption sites compared to Cr(VI) ions was higher leading to higher Cr(VI) removal, but as the initial concentration of Cr(VI) was increased, this ratio (adsorption site/Cr(VI) ion) decreased resulting in saturation, lesser %*R*, and higher adsorption capacities [25, 36, 46].

To account for the interaction between Cr(VI) and MHPD adsorbents, nonlinear Langmuir and Freundlich adsorption isotherms were used to model the adsorption equilibrium data from the effect of concentration. The Langmuir pertains to monolayer adsorption of adsorbates onto homogeneous adsorption sites, while the Freundlich advocates the multilayer adsorption mechanism on heterogeneous sites.  Table 3 illustrates the adsorption constants obtained from the isotherms. Comparing the MHP adsorbents using the coefficient of determination (*R*^2^) and the variance, it can be seen that the Freundlich model exhibited higher *R*^2^ values and lower variance. In addition, the *q*_e_ and Langmuir *q*_m_ values were not closer together. Therefore, it was concluded that the data for MHP were best described by the Freundlich model, implicating that the mechanism of removal was a multilayer process on heterogeneous adsorption sites. The Freundlich 1/*n*_F_ values were less than unity indicating a feasible and favorable adsorption of Cr(VI) on the MHP surface.

#### 3.2.6. Effect of Dosage and Adsorption Capacity Comparison

**Figure 10:**
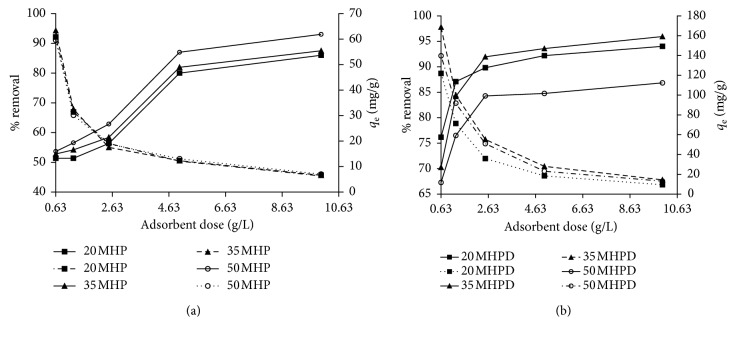
Effect of adsorbent dosage for the removal of Cr(VI) by MHP and MHPD adsorbents (conditions: initial Cr(VI) concentration 150 mg/L; pH 2; and time 120 min).

Figures 10(a) and 10(b) portray the %*R* of Cr(VI) as a function of adsorbent dosage for MHP and MHPD adsorbents. In both cases, the %*R* of Cr(VI) increased with increasing dose levels from 0.63 to 10.63 g/L. The %*R* increased sharply from 50 to 88% as the dose was increased from 0.63 to 4.63 g/L and slowed down afterward almost reaching equilibrium for MHP adsorbents at 92% removal. For MHPD adsorbents, the %*R* increased steeply from 67 to 93% when the dosage was increased from 0.63 to 2.63 g/L and only increased to 95% afterward as equilibrium was attained. It is clear that MHPD adsorbents were superior to MHP adsorbents as only half the dosage (2.63 g/L) was needed to achieve the same maximum %*R* by MHP at 4.63 g/L. The increased removal at higher doses could be due to the presence of more adsorption sites leading to high ion exchange capacity and surface area [5, 34, 36]. In both cases, the adsorption capacity decreased as the dose was increased probably because there was overlapping resulting from overcrowding of adsorption sites [47].

The performance of the prepared adsorbents was demonstrated by comparing their adsorption capacities to those of other biosorbents found in the literature. The results illustrated in Table 4 revealed that adsorption capacities of adsorbents may vary widely making it difficult to compare because of different experimental conditions (dosage, pH, adsorbate concentration, stirring rate, shaking versus stirring, and column versus batch) used. Nonetheless, the present result was comparable to other adsorbents but lower than others.

### 3.3. Selectivity of Cr(VI) Ion

**Figure 11:**
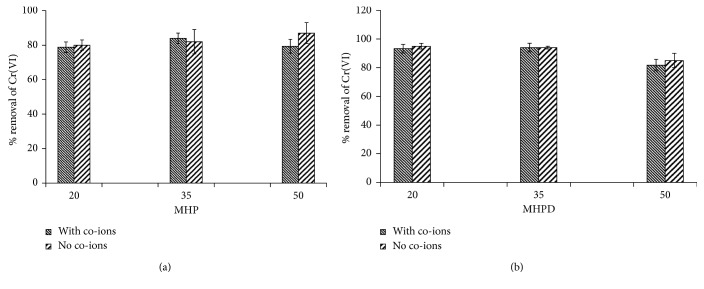
The effect of co-ions on the removal of Cr(VI) by MHP (a) and MHPD (b) adsorbents.

Investigation of the effect of competing ions is paramount for any developed adsorbent because pollutants often exist together with a host of other chemicals including organic, inorganic, and biological materials. It has been shown that the presence of sulphates and nitrates impacted the removal of Cr(VI) negatively [15, 48, 49]. In this study, a concoction of a solution containing 150 mg/L of each of Cr(VI), SO_4_^2−^, Cl^−^, and NO_3_^−^ was prepared from their respective salts and used to investigate the effect of co-ions on adsorption of Cr(VI). Another solution containing only Cr(VI) was used as a control. The results depicting the %*R* of Cr(VI) in the presence and absence of co-ions are presented in Figure 11. The percent removal of Cr(VI) decreased from 80 to 79% for 20 MHP, 87 to 80% for 50 MHP, 95 to 93% for 20 MHPD, and 85 to 82% for 50 MHPD, while the %*R* was exactly the same for 35 MHPD but the removal was higher in the presence of co-ions when 35 MHP was used. The 50 MHPD adsorbents showed superior selectivity than 20 MHPD and 35 MHPD. The latter was negatively affected by the presence of competing ions, but still, the %*R* was still greater than 80%. The prepared adsorbents demonstrated good recognition of Cr(VI) in the presence of competing ions and can thus be recommended as alternative low-cost adsorbents for the mitigation of Cr(VI).

### 3.4. Application to Real-World Sample

**Figure 12:**
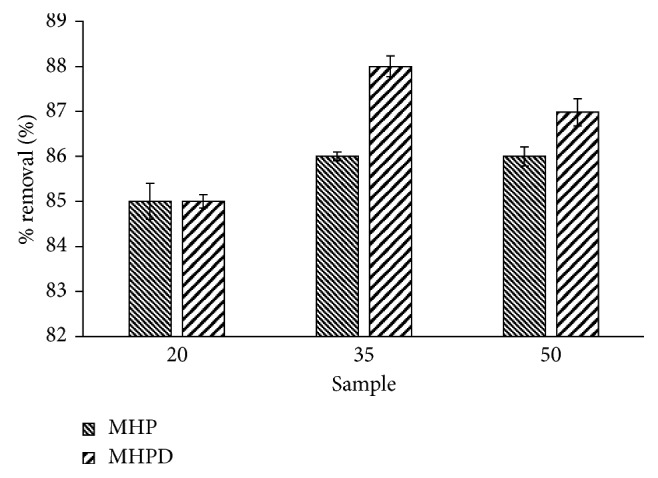
Real sample characteristics and removal of Cr(VI) from the real sample by MHP and MHPD adsorbents.

A wastewater sample with the chemical qualities Cr^3+^ (4 mg/L), Zn^2+^ (1 mg/L), Fe^2+^ (6 mg/L), Ni^2+^ (2 mg/L), and Cu^2+^ (31 mg/L) was used to investigate the performance of the developed adsorbents in a real-world sample. All were prepared from their chloride salts. The sample was spiked with 30 mg/L Cr(VI).  Figure 12 shows the results of %*R* of Cr(VI) from the spiked solution by MHPD adsorbents. The efficiency in the removal of Cr(VI) by MHP adsorbents decreased in the order 20 MHP > 35 MHP > 50 MHP from 82%, 75%, and 53% removal, respectively. In contrast, the removal increased in the order 80%, 85%, and 88% for 20 MHPD, 35 MHPD, and 50 MHPD, respectively. The decrease in %*R* as the percent of H_2_O_2_ increased (20 MHP, 35 MHP, and 50 MHP) was probably due to the high density of oxygenated groups on the surface imparted by a high concentration of H_2_O_2_ leading to the high affinity for cations. On the other hand, the removal increased with increasing H_2_O_2_ concentration for MHPD because of higher concentration of DPC which favored removal of Cr(VI) at low pH due to protonation. The results revealed that the prepared adsorbents are suitable to be used for the remediation of water contaminated with metal ions as the %*R* was greater than 70% except for the 50 MHP adsorbents.

## 4. Conclusion

Three different concentrations of hydrogen peroxide (20, 35, and 50% vol.) were utilized to pretreat the *Macadamia* nutshell powder. The pretreatment imparts some oxygen-containing groups (C=O, OH, and C–O–C) on the surface of the nutshells. It was demonstrated by FTIR that the H_2_O_2_ treatment resulted in shifting of C=O peak from 1712 to 1728 cm^−1^ and the C–O peak from 1031 to 1029 cm^−1^ in comparison to the RMN. Grafting of 1,5′-diphenylcarbazide on the RMN was confirmed with FTIR, SEM, and TGA. The FTIR showed new peaks at 3333 and 1657 cm^−1^ attributed to NH and amide C=O, respectively. Successful grafting was further corroborated by the TGA curves of MHPD showing high volatility due to the presence of DPC compared to MHP. SEM micrographs of MHPD exhibited much rougher surface than the MHP, and this was associated with grafting. The BET surface area ranged from 0.0063 to 0.5093 m^2^/g, and these were typical of biomass materials. The adsorption efficiency of MHP treated with 50% H_2_O_2_ improved from 37.74 to 72.12 mg/g for the grafted materials. The improvement in adsorption capacity validated the efficiency of grafting. The adsorption process was best described by Langmuir and PSO. In addition, it was shown that intraparticle diffusion was not the only rate-controlling step. The adsorption of Cr(VI) by MHPD was less affected by the presence of competing ions as it was shown in selectivity studies and application to real-world sample.

## Figures and Tables

**Table 1:** BET surface characterization.

Adsorbents	Surface characterization		Pore size (nm)
	Pore volume (cm^3^/g)	BET surface area (m^2^/g)	
20 MHP	—	0.0063	—
35 MHP	0.002717	1.0019	10.85013
50 MHP	—	0.5093	—
20 MHPD	—	0.1047	—
35 MHPD	—	0.2034	—
50 MHPD	—	0.0647	—

**Table 2:** PFO and PSO kinetic parameters for adsorption of Cr(VI) by MHP and MHPD.

Parameters	20 MHP	35 MHP	50 MHP	20 MHPD	35 MHPD	50 MHPD
*q*_t_ (mg/g)	18.11	18.96	22.5	58.77	56.89	56.81
*q*_e_ (mg/g)	21.26	17.05	21.77	57.96	55.80	53.58
*k*_1_ (1/min)	0.0107	0.0224	0.0325	0.1286	0.0644	0.151
*R* ^2^	0.939	0.615	0.940	0.799	0.967	0.637
*X* ^2^	41.71	14.04	11.24	0.40	5.93	0.780
*q*_e_ (mg/g)	31.09	20.82	25.73	59.52	60.25	56.78
*k*_2_ (g/mg min)	0.0025	0.00117	0.00147	0.0085	0.00198	0.00863
*R* ^2^	0.942	0.725	0.936	0.896	0.881	0.887
*X* ^2^	89.12	26.83	22.1	0.95	11.72	2.19

**Table 3:** Freundlich and Langmuir adsorption constants for MHP.

Models	Parameters	20 MHP	35 MHP	50 MHP
	*q*_e_ (mg/g)	14.60	22.24	37.74
Langmuir	*q*_m_ (mg/g)	21.77	27.76	60.59
*q*_e_=*q*_max_ · *b* · *C*_e_/(1+*bC*_e_)	*b* (L/mg)	0.0173	0.0391	0.0189
	*R* ^2^	0.873	0.935	0.961
	Var^∗^	2.72	2.81	6.67

	*K*_F_ (mg^1−1*n*^L^1/*n*^/g)	1.33	3.27	3.39
Freundlich	*n*_F_	1.96	2.33	1.84
*q*_e_=*K*_F_ · *C*_e_^1/nf^	*R* ^2^	0.906	0.947	0.974
	Var^∗^	2.02	2.29	4.39

^∗^Sum of square errors divided by degrees of freedom.

**Table 4:** Comparison of adsorption capacities.

Adsorbents	Pretreatment	Functionalization	pH	*q*_m_ (mg/g)	References
Potato peels	HCl	—	2.5	3.28	[45]
Raw rutin	Extraction/isolation	—	3	26.3	[34]
Rutin resin	Formaldehyde/HNO_3_	—	3	41.6	[34]
Banana peels	HCl/NaOH/H_2_O_2_	Acrylonitrile grafted	3	6.17	[31]
Palm branches	H_2_SO_4_	—	2	25	[32]
Palm branches	Acetic acid	Chitosan	6	55	[32]
Palm branches	H_2_SO_4_	Cationic surfactant	6	41.7	[32]
Grape peelings	H_2_O_2_	—	5.5	39.06	[7]
Banana peels	—	—	5	3	[15]
Orange peels	—	—	3	9	[15]
Coir pith	—	Acrylic acid	2	165	[16]
Masau stones	NaOH	Epichlorohydrin/diethylenetriamine	3.5	87.33	[19]
Coir pith	—	Acrylic acid	2	196	[16]
*Macadamia* nutshell	H_2_O_2_	—	1	37.74	This study
*Macadamia* nutshell	H_2_O_2_	Diphenylcarbazide	1	72.12	This study

## Abbreviations

DPC: 1,5′-diphenylcarbazide
MHP: *Macadamia* nutshell powder oxidized by hydrogen peroxide solutions
20 MHP: *Macadamia* nutshell powder oxidized by 20% (v/v) hydrogen peroxide solutions
35 MHP: *Macadamia* nutshell powder oxidized by 35% (v/v) hydrogen peroxide solutions
50 MHP: *Macadamia* nutshell powder oxidized by 50% (v/v) hydrogen peroxide solutions
20 MHPD: 20 MHP adsorbent grafted with DPC
35 MHPD: 35 MHP adsorbent grafted with DPC
50 MHPD: 50 MHP adsorbent grafted with DPC
BET: Brunauer–Emmett–Teller
DTA: Differential thermal analysis
EDX: Energy dispersive X-ray
FTIR: Fourier transform infrared spectroscopy
LOD: Limit of detection
PFO: Pseudo-first order
PSO: Pseudo-second order
RMN: Raw *Macadamia* nutshell
SEM: Scanning electron microscopy
TGA: Thermogravimetric analysis.

## Additional Points

*Highlights*. *Macadamia* nutshell powder was oxidized with hydrogen peroxide. The peroxide-modified materials were grafted with diphenylcarbazide. Grafting with diphenylcarbazide improved the adsorption capacity. Presence of co-ions did not influence the removal efficiency. Adsorbents demonstrated good recoveries when subjected to real-world sample.
